# Supplementary material for: Content-rich biological network constructed by mining PubMed abstracts
Source: BMC Bioinformatics. 2004 Oct 8;5:147. doi: 10.1186/1471-2105-5-147 (PMC528731; doi:10.1186/1471-2105-5-147)
Supplement: Additional File 2 — The original results of the above study (non-essential files are deleted to keep the file size under the limit set by BMC bioinformatics). [file 1471-2105-5-147-S2.bz2 › chilibotAdditionalFile2/dip10/588ID1303763E10959/html/PYRB_PYRI.html]

 


 **PYRB** and **PYRI** 
  
Found 17 abstracts in PubMed, retrieved 10.  
 

 What does Google say? 
 PDF only 
| .edu only 

---

**Interactive relationship** (e.g. stimulation, inhibition, etc)

**Neutral relationship**- The two genes were found to constitute an operon, with  **pyrI**  being the distal gene and separated from  **pyrB**  by a 15 bp intercistronic region.  Ref: 3036524 Eur J Biochem, 1987
- The genes constitute a pyrBI  [ **PYRB** ]  operon homologous to the cognate structure in E. coli where  **pyrB**  and  **pyrI**  respectively encode the catalytic and the regulatory chains of ATCase  [ **PYRB** ] .  Ref: 9611817 Microbiology, 1998
- The plasmid with appropriate stop codons also contained  **pyrI** , the gene encoding the regulatory chain of ATCase  [ **PYRB** ] .  Ref: 8876180 Proc Natl Acad Sci U S A, 1996
- We now report the sequence of the mutated  **pyrI**  and show that, during the generation of this pyrBI  [ **PYRB** ]  bearing phage, six codons from lambda DNA have been substituted for the eight terminal codons of the wild type gene.  Ref: 3912513 J Mol Biol, 1985

**Non-interactive relationship** (e.g. studied together, co-existance, homology, etc.)

- A purification protocol was devised for the Mj  **PyrB**  and M. jannaschii  **PyrI**  Mj  **PyrI**  gene products.  Ref: 10748118 J Biol Chem, 2000
- The  **pyrB**   **pyrI**  region of the Salmonella typhimurium LT2 chromosome has been cloned and sequenced.  Ref: 3036524 Eur J Biochem, 1987
- The plasmid used for the over expression carries the gene for the regulatory subunit,  **pyrI** , downstream from the strong  **pyrB**  promoter.  Ref: 8433966 Protein Eng, 1993
- The deduced amino acid sequences of the  **pyrB**  and  **pyrI**  genes showed 27.6 50% identity with archaeal and enterobacterial ATCases.  Ref: 10447693 Eur J Biochem, 1999
- Comparison of primary sequences of the polypeptides encoded by the  **pyrB**  and  **pyrI**  genes with those of homologous eubacterial and eukaryotic chains showed a high degree of conservation of the residues which in E. coli ATCase  [ **PYRB** ]  are involved in catalysis and allosteric regulation.  Ref: 9209027 J Bacteriol, 1997
- The genes encoding the catalytic  **pyrB**  and regulatory  **pyrI**  polypeptides of aspartate transcarbamoylase ATCase  [ **PYRB** ] , EC 2.1.3.2 from several members of the family Enterobacteriaceae appear to be organized as bicistronic operons.  Ref: 3722124 J Bacteriol, 1986
- Homology models of the Mj  **PyrB**  and Mj  **PyrI**  gene products were constructed based on the three dimensional structures of the homologous E. coli proteins.  Ref: 10748118 J Biol Chem, 2000
- Molecular weight measurements of the Mj  **PyrB**  and Mj  **PyrI**  gene products revealed that the Mj  **PyrB**  gene product is a trimer and the Mj  **PyrI**  gene product is a dimer.  Ref: 10748118 J Biol Chem, 2000
